# Development and validation of a machine learning model for predicting postherpetic neuralgia risk

**DOI:** 10.3389/fneur.2026.1775957

**Published:** 2026-04-16

**Authors:** Xiao Chen, Xinqiang Lin, Chaoyue Lin, Changgui Lin, Jian Lin, Jiabin Li

**Affiliations:** 1 Department of Pain Medicine, The Affiliated Hospital of Putian University, Putian, China; 2 Department of Dermatology, The Affiliated Hospital of Putian University, Putian, China; 3 Department of Neurology, The Affiliated Hospital of Putian University, Putian, China; 4 Department of Anesthesiology, Zhangzhou Affiliated Hospital of Fujian Medical University, Zhangzhou, China

**Keywords:** herpes zoster, machine learning, postherpetic neuralgia, risk prediction, XGBoost

## Abstract

**Objective:**

Postherpetic neuralgia (PHN) is a debilitating complication of herpes zoster (HZ), and early identification of high-risk patients is crucial for timely intervention. This study aimed to develop and validate machine learning models to predict the risk of developing PHN following HZ onset.

**Methods:**

A retrospective analysis of two prospective cohorts was performed. The training cohort comprised 627 patients from the Affiliated Hospital of Putian University, and an independent external validation cohort included 219 patients from Zhangzhou Affiliated Hospital of Fujian Medical University. Least Absolute Shrinkage and Selection Operator (LASSO) regression and the Boruta algorithm were used for feature selection. Ten ML models were constructed and evaluated based on metrics including the area under the receiver operating characteristic curve (AUC), accuracy, sensitivity, F1 score, calibration, and clinical utility. The optimal model was further interpreted using SHapley Additive exPlanations (SHAP).

**Results:**

The incidence of PHN was 19.0% (119 of 627) in the training cohort and 22.8% (50 of 219) in the validation cohort. Subsequently, five key risk factors were identified. Among the 10 models, XGBoost exhibited the best comprehensive performance, with an AUC of 0.826 (95%CI: 0.786–0.866) in the training cohort and 0.840 (95%CI: 0.784–0.896) in the validation cohort. SHAP analysis revealed that age was the most important predictor, followed by timing of antiviral therapy, acute pain severity, prodromal phase pain, and diabetes.

**Conclusion:**

The XGBoost model based on five clinically accessible factors effectively predicts the risk of PHN. This tool can assist clinicians in early risk stratification and guide personalized management for patients with HZ.

## 1 Introduction

Herpes zoster (HZ) is an acute infectious dermatosis resulting from the reactivation of the varicella-zoster virus (VZV) dormant in dorsal root ganglia. It is characterized by unilateral, dermatomal vesicular eruptions and severe neuropathic pain (1, 2). HZ is a global health concern, with an incidence that rises markedly with age. The annual incidence ranges from approximately 3.8 to 12.2 per 1,000 adults, increasing sharply in older populations (3–5).

Timely and standardized pharmacotherapy is the cornerstone of acute HZ management. The primary goals are to alleviate acute pain, shorten the duration of skin lesions, and reduce the risk of developing Postherpetic Neuralgia (PHN) (6). First-line treatment typically involves antiviral agents combined with analgesic medications (7). However, even with strict adherence to clinical guidelines and prompt initiation of therapy, the incidence of PHN remains substantial, affecting approximately 2.6–46.7% of HZ patients (8–10). PHN is characterized by persistent neuropathic pain that can endure for months to years, leading to significant morbidity such as burning sensations, allodynia, sleep disturbances, depression, impaired quality of life, and increased healthcare utilization and economic burden (11–14).

Early interventional therapies, such as nerve blocks, pulsed radiofrequency ablation, and spinal cord stimulation, are effective in alleviating pain and reducing the risk of PHN in high-risk patients (15–18). However, these procedures are invasive, are associated with potential complications, and impose a substantial economic burden. Therefore, the accurate identification of patients at high risk for PHN is of critical clinical importance, as it enables a targeted management approach: high-risk patients may benefit from combined pharmacological and interventional strategies, while low-risk individuals can be spared unnecessary invasive procedures.

Although several prediction models for PHN have been developed, they are frequently limited by small sample sizes, lack of external validation, suboptimal predictive accuracy, and insufficient generalizability (10, 19–23). To address these limitations, this study aimed to develop and rigorously validate a novel machine learning model using a dual-cohort design. Our objective was to provide clinicians with a practical and interpretable tool for early risk stratification to facilitate informed decision-making regarding preventive interventions for patients with HZ.

## 2 Methods

### 2.1 Patients and study design

We conducted a retrospective analysis using data from a prospectively maintained database at the Affiliated Hospital of Putian University (September 2021 to May 2025). The inclusion criteria were: (1) confirmed diagnosis of HZ; (2) age ≥ 18 years; and (3) receipt of standardized antiviral and analgesic pharmacotherapy. Exclusion criteria included: incomplete 90-day follow-up data; recent acute severe infection; receipt of interventional therapies (e.g., nerve block, radiofrequency ablation, or spinal cord stimulation) within 3 months of onset; and presence of psychiatric disorders, neurological diseases, or other conditions that could interfere with somatic sensation assessment.

**Figure 1:**
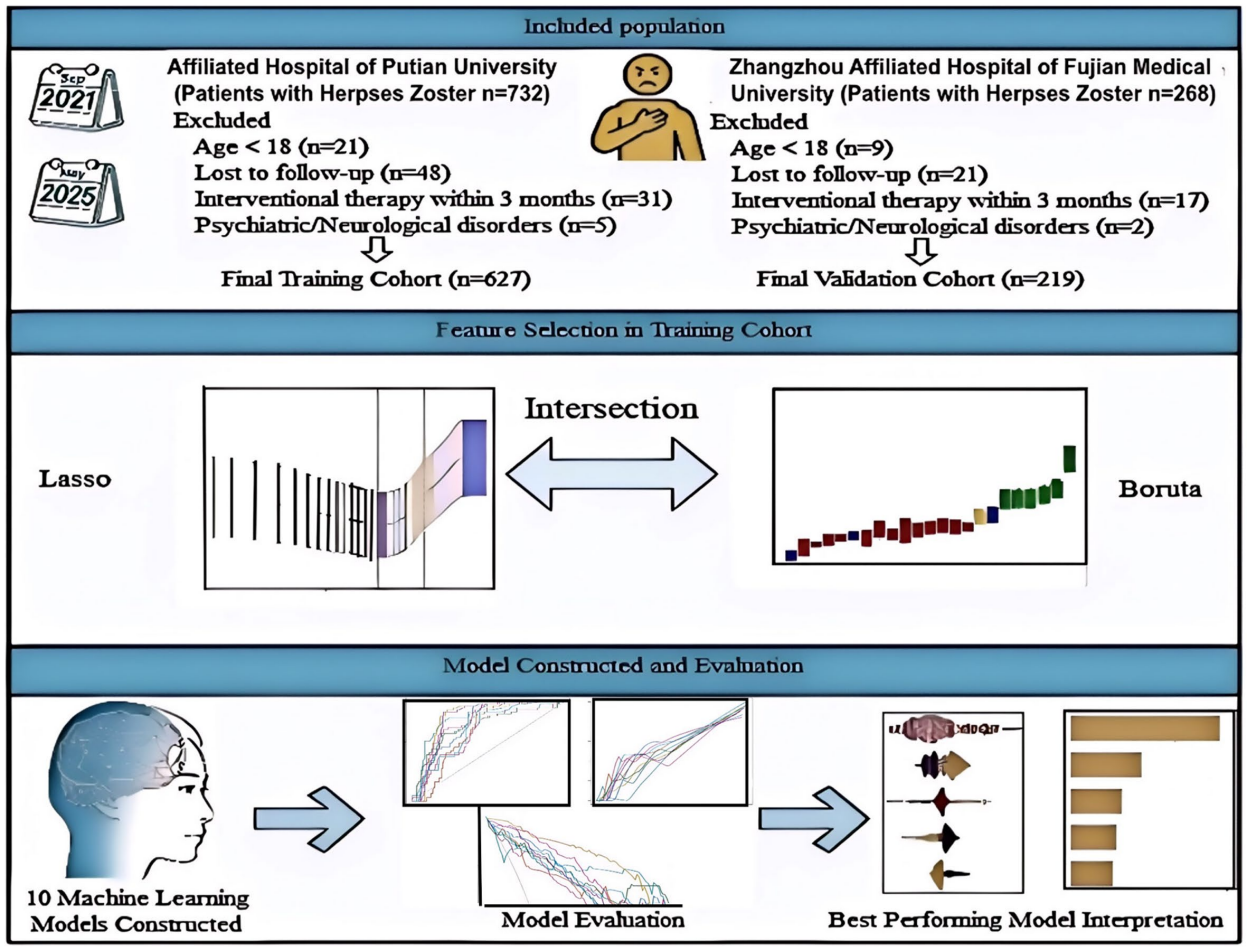
Study flowchart.

A total of 627 eligible patients were included as the training cohort. Applying the same criteria, 219 patients from Zhangzhou Affiliated Hospital of Fujian Medical University were enrolled as an independent external validation cohort. The patient selection process is summarized in Figure 1.

### 2.2 Observation indicators and definitions

PHN was diagnosed in cases of persistent neuropathic pain and sensory disturbance persisting beyond 3 months after the onset of HZ infection (24, 25). Follow-up for PHN assessment was conducted by telephone interview at 3 months post-infection.

For the purpose of this study, chronic respiratory diseases encompassed three major conditions: asthma, chronic bronchitis, and chronic obstructive pulmonary disease (COPD) (26, 27). Immunosuppression was defined as the presence of leukemia, lymphoma, HIV infection, myeloma, a history of hematopoietic stem cell transplantation, other clinically significant cellular immune deficiencies, or the administration of high-dose oral corticosteroids during the acute neuralgia phase (23, 28).

Acute pain severity at initial diagnosis was assessed using the Numeric Rating Scale (NRS), an 11-point scale ranging from 0 (no pain) to 10 (worst imaginable pain). Scores were categorized as mild (1–3), moderate (4–6), or severe (7–10) (29–31).

### 2.3 Statistical methods

Statistical analyses were performed using SPSS (version 27.0) and R (version 4.5.2). Categorical variables were compared using the *χ*^2^ test. The normality of continuous variables was assessed using the Shapiro–Wilk test. Normally distributed variables were compared using the Student’s *t*-test, and non-normally distributed variables using the Mann–Whitney *U* test. A two-tailed *p*-value < 0.05 was considered statistically significant.

In the training cohort, feature selection was performed using Least Absolute Shrinkage and Selection Operator (LASSO) regression and the Boruta algorithm. We employed both methods to enhance robustness: LASSO provides a regularized, parsimonious subset of predictors, while Boruta evaluates feature importance via random forest permutations. This dual approach helps mitigate limitations inherent in any single method and increases confidence in the selected features. Subsequently, 10 machine learning models were constructed based on the selected features: random forest (RF), extreme gradient boosting (XGBoost), support vector machine (SVM), artificial neural network (ANN), logistic regression (LR), light gradient boosting machine (LightGBM), adaptive boosting (AdaBoost), naive Bayes (NB), k-nearest neighbors (KNN), and decision tree (DT). Hyperparameter optimization was performed for each model using grid search with 5-fold cross-validation. The search grids were tailored to each algorithm based on common practice and computational feasibility. Full hyperparameter search spaces are provided in Supplementary Table 1. Model performance was evaluated and compared using metrics including the area under the receiver operating characteristic curve (ROC), accuracy, sensitivity, specificity, positive predictive value (PPV), and F1 score. The optimal model was further interpreted using SHapley Additive exPlanations (SHAP).

### 2.4 Model training environment

All machine learning model training and evaluation procedures were conducted on a local computer. The hardware specifications and computational time required for the complete training process are detailed in Supplementary Table 2.

## 3 Results

### 3.1 Patient characteristics and PHN incidence in training and validation cohorts

The baseline characteristics of patients in the training cohort (*n* = 627) and the external validation cohort (*n* = 219) are presented in Table 1. The incidence of PHN at 90 days was 19.0% (119 of 627) in the training cohort and 22.8% (50 of 219) in the validation cohort.

**Table 1:** Comparison of baseline characteristics between the training and validation cohorts.

Variable	Training cohort (*n* = 627)	Validation cohort (*n* = 219)	*P*-value
Gender			0.243
Male	292 (46.6)	92 (42.0)	
Female	335 (53.4)	127 (58.0)	
Age (years), mean (±SD)	60.8 ± 11.8	61.7 ± 11.3	0.320
Smoking history			0.378
Yes	147 (23.4)	45 (20.5)	
No	480 (76.6)	174 (79.5)	
Alcohol consumption			0.033
Yes	178 (28.4)	46 (21.0)	
No	449 (71.6)	173 (79.0)	
Surgical history			0.464
Yes	112 (17.9)	44 (20.1)	
No	515 (82.1)	175 (79.9)	
Diabetes			0.441
Yes	192 (30.6)	61 (27.9)	
No	435 (69.4)	158 (72.1)	
Hypertension			0.159
Yes	202 (32.2)	82 (37.4)	
No	425 (67.8)	137 (62.6)	
Hyperlipidemia			0.502
Yes	248 (39.6)	81 (37.0)	
No	379 (60.4)	138 (63.0)	
Chronic respiratory diseases			0.169
Yes	92 (14.7)	24 (11.0)	
No	535 (85.3)	195 (89.0)	
Immunosuppression			0.596
Yes	56 (8.9)	17 (7.8)	
No	571 (91.1)	202 (92.2)	
Under neoplasm treatment			0.496
Yes	52 (8.3)	15 (6.8)	
No	575 (91.7)	204 (93.2)	
BMI (kg/m^2^), mean (±SD)	22.9 ± 3.0	23.3 ± 3.1	0.098
Prodromal phase pain			
Yes	408 (65.1)	136 (62.1)	0.429
No	219 (34.9)	83 (37.9)	
Acute pain severity			0.606
Mild	110 (17.5)	34 (15.5)	
Moderate	426 (67.9)	148 (67.6)	
Severe	91 (14.5)	37 (16.9)	
Affected side			0.191
Left	307 (49.0)	96 (43.8)	
Right	320 (51.0)	123 (56.2)	
Site of involvement			0.310
Head and neck	104 (16.6)	33 (15.1)	
Trunk	482 (76.9)	165 (75.3)	
Limbs	41 (6.5)	21 (9.6)	
White blood cell count (×10^9^/L), median (IQR)	6.25 [4.90, 7.50]	5.98 [4.80, 7.19]	0.078
Hemoglobin (g/L), median (IQR)	127.00 [115.00, 137.00]	129.00 [117.00, 139.00]	0.192
Platelet count (×10^9^/L), median (IQR)	231.00 [189.00, 267.00]	243.00 [201.50, 278.00]	0.015
Timing of antiviral therapy (days)			0.483
≤3	312 (49.8)	115 (52.5)	
>3	315 (50.2)	104 (47.5)	

BMI, body mass index.

Most baseline clinical and demographic characteristics were comparable between the two cohorts (*p* > 0.05). Significant differences were observed only in the proportion of patients with a history of alcohol consumption (*p* = 0.033) and in the median platelet count, which was higher in the validation cohort (*p* = 0.015). Overall, the two cohorts demonstrated substantial similarity in baseline profiles.

### 3.2 Variable selection

**Figure 2:**
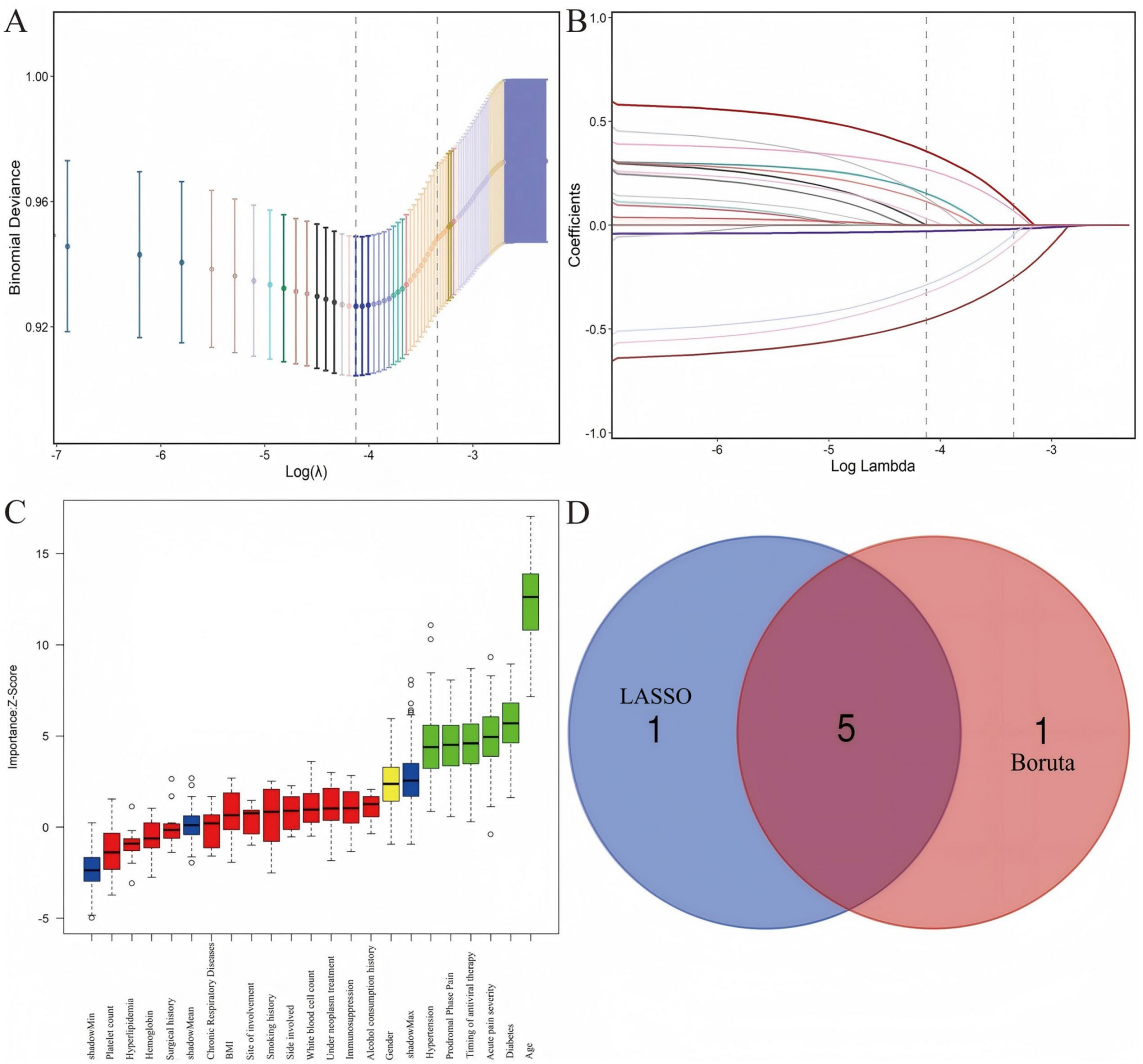
Variable selection for PHN predictors. **(A)** Binomial deviance curve of the LASSO regression. **(B)** Coefficient paths of the LASSO regression. **(C)** Features confirmed as important by the Boruta algorithm are shown in green, rejected (non-informative) features in red, tentative features in yellow, and shadow (randomized) features in blue. **(D)** Venn diagram illustrating the intersection of factors selected by LASSO and Boruta.

In the training cohort, both LASSO regression and the Boruta algorithm were employed to identify potential predictors of PHN. Using the one-standard-error rule (*λ*₁ₛₑ = 0.0363), LASSO regression identified six predictors. The Boruta algorithm also selected six features. The intersection of these two sets yielded five common variables: age, diabetes, prodromal pain, acute pain severity, and timing of antiviral therapy, as illustrated in Figure 2.

### 3.3 Model development and performance evaluation

**Figure 3:**
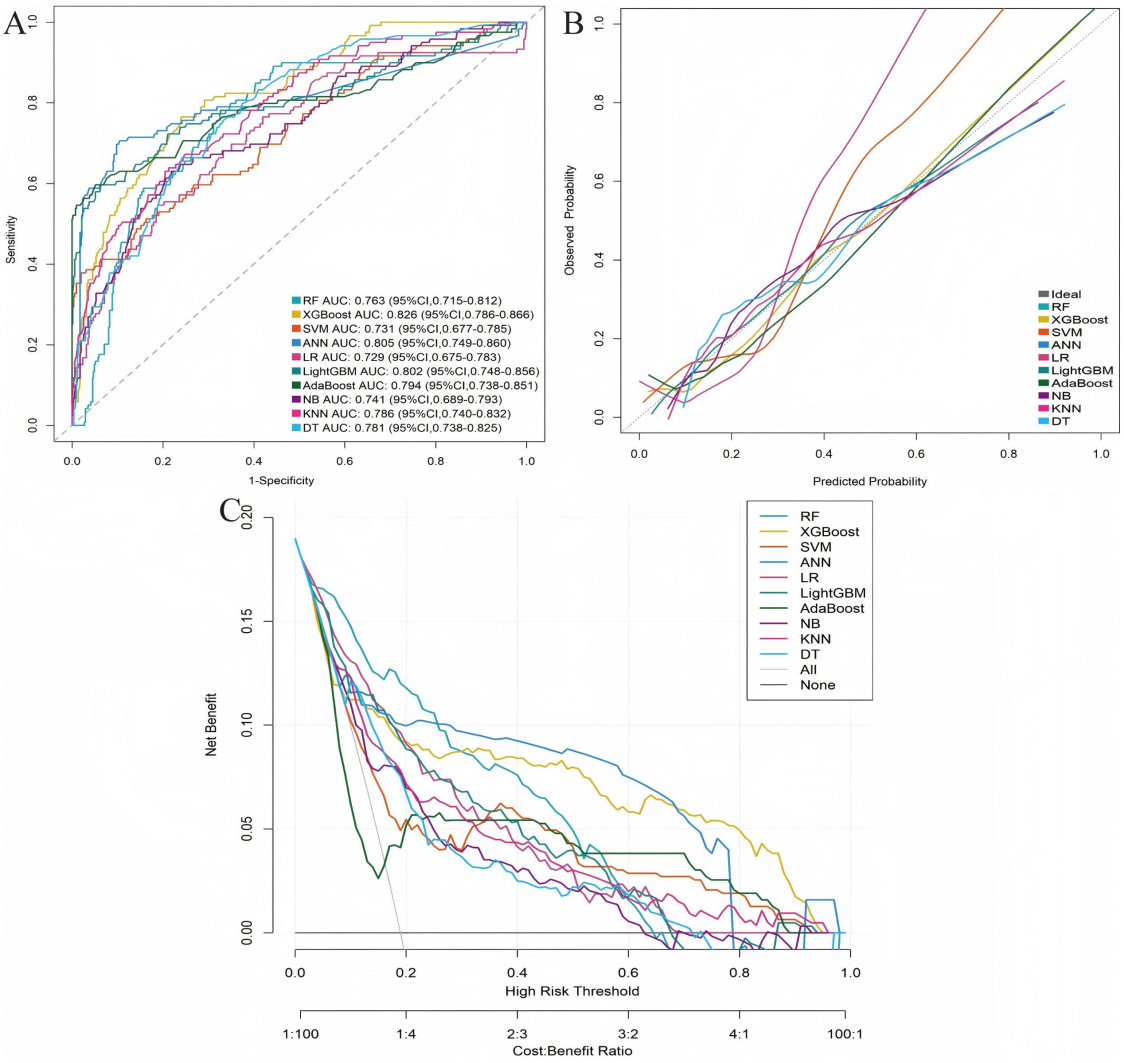
Model comparison in training cohort. **(A)** ROC curves. **(B)** Calibration plots. **(C)** Decision curves.

**Figure 4:**
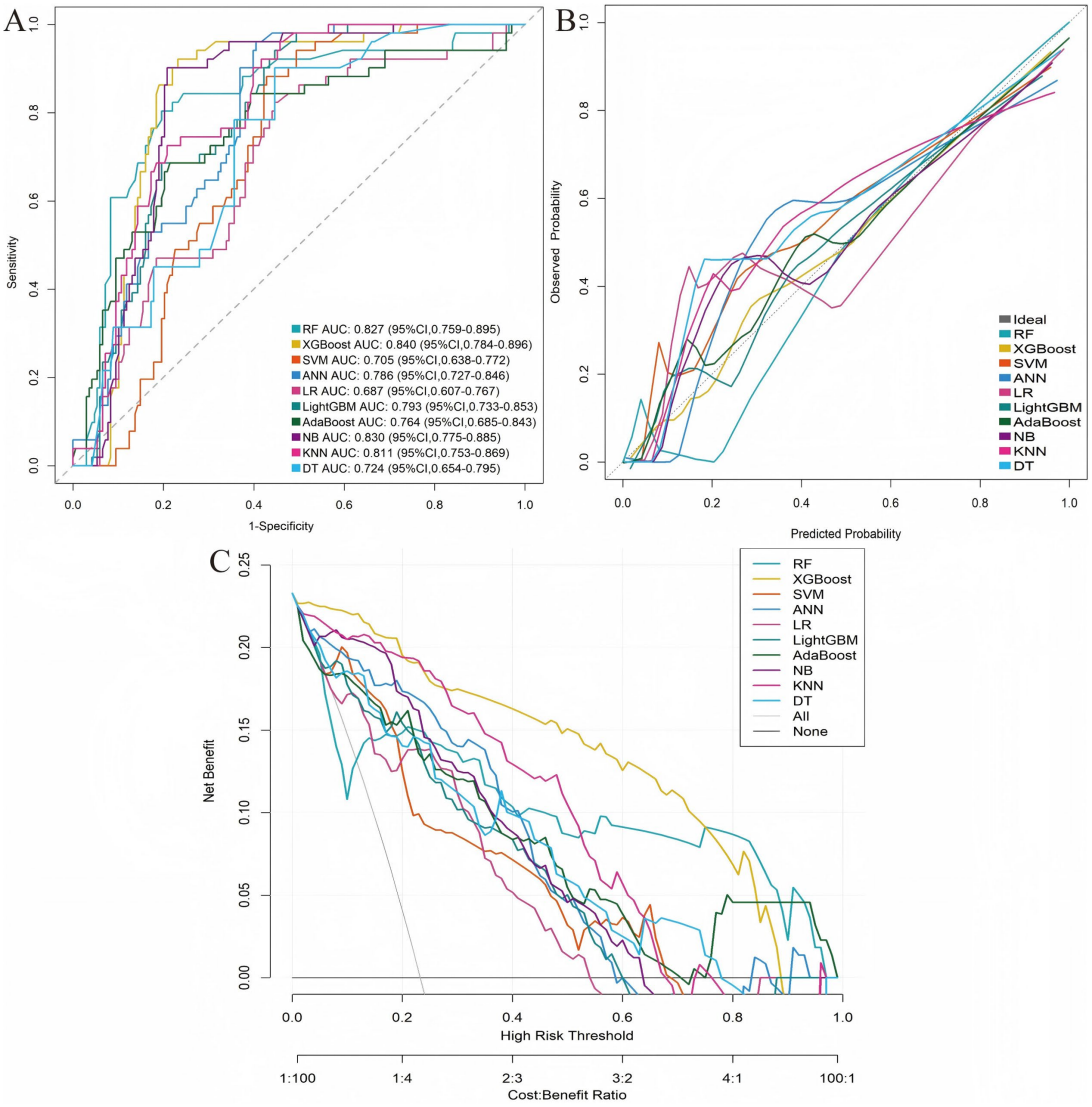
Model comparison in validation cohort. **(A)** ROC curves. **(B)** Calibration plots. **(C)** Decision curves.

Based on the five identified risk factors, 10 machine learning models were developed to predict PHN. Performance metrics are summarized in Table 2. Among all models, XGBoost demonstrated the best overall predictive performance. The ROC curves, calibration plots, and decision curve analysis (DCA) for both cohorts are presented in Figures 3, 4, which collectively substantiate the superior and consistent performance of the XGBoost model.

**Table 2:** Performance metrics of 10 machine learning models for predicting PHN.

Model	AUC	Accuracy	Sensitivity	Specificity	PPV	F1 score
Training cohort						
RF	0.763 (0.715–0.812)	0.679	0.798	0.652	0.349	0.486
XGBoost	0.826 (0.786–0.866)	0.861	0.765	0.860	0.727	0.548
SVM	0.731 (0.677–0.785)	0.866	0.378	0.980	0.818	0.517
ANN	0.805 (0.749–0.860)	0.860	0.706	0.896	0.613	0.656
LR	0.729 (0.675–0.783)	0.761	0.546	0.811	0.404	0.464
LightGBM	0.802 (0.748–0.856)	0.872	0.597	0.937	0.689	0.640
AdaBoost	0.794 (0.738–0.851)	0.882	0.597	0.949	0.732	0.658
NB	0.741 (0.689–0.793)	0.753	0.630	0.781	0.403	0.492
	0.786 (0.740–0.832)	0.759	0.639	0.787	0.413	0.502
DT	0.781 (0.738–0.825)	0.659	0.798	0.626	0.333	0.470
Validation cohort						
RF	0.827 (0.759–0.895)	0.804	0.804	0.804	0.554	0.656
XGBoost	0.840 (0.784–0.896)	0.804	0.922	0.768	0.547	0.687
SVM	0.705 (0.638–0.772)	0.644	0.882	0.571	0.385	0.536
ANN	0.786 (0.727–0.846)	0.680	0.961	0.595	0.419	0.584
LR	0.687 (0.607–0.767)	0.616	0.824	0.554	0.359	0.500
LightGBM	0.793 (0.733–0.853)	0.644	0.941	0.554	0.390	0.551
AdaBoost	0.764 (0.685–0.843)	0.763	0.686	0.786	0.493	0.574
NB	0.830 (0.775–0.885)	0.817	0.902	0.792	0.568	0.697
KNN	0.811 (0.753–0.869)	0.776	0.725	0.792	0.514	0.602
DT	0.724 (0.654–0.795)	0.635	0.902	0.554	0.380	0.535

### 3.4 XGBoost interpretability by SHAP

**Figure 5:**
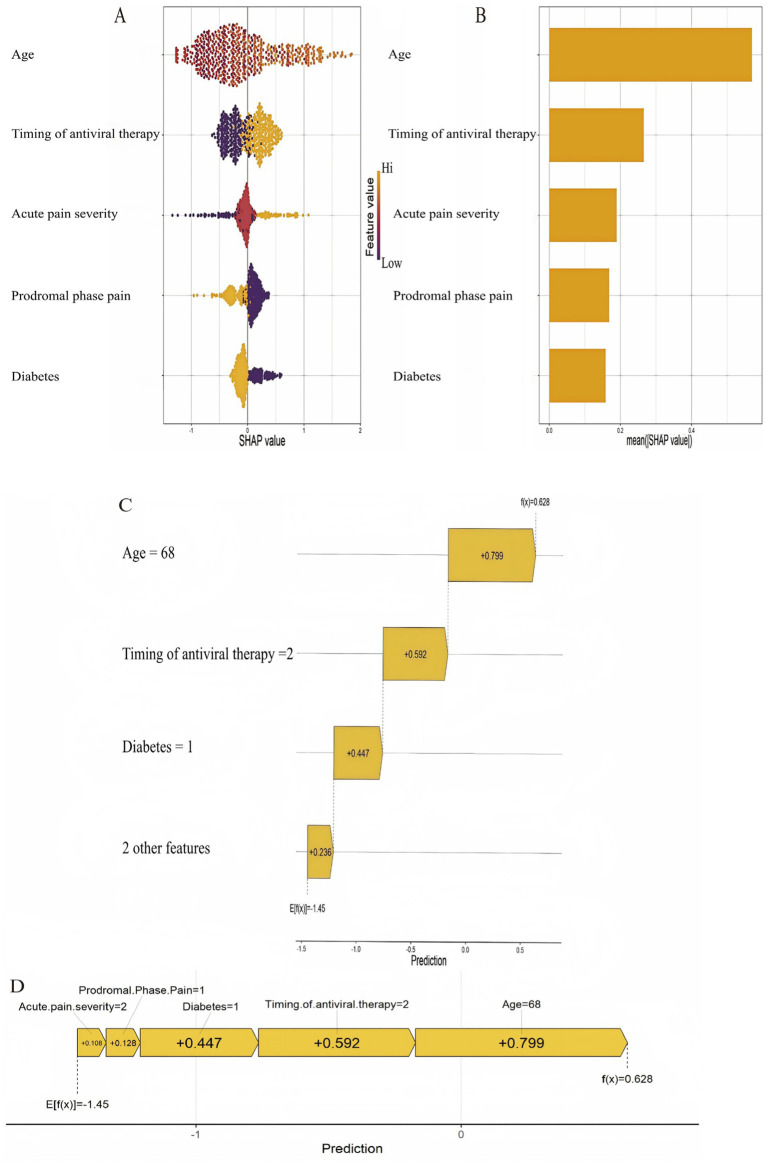
SHAP-based feature importance analysis for the XGBoost model. **(A)** SHAP bee swarm plot showing the feature value distribution and impact direction. **(B)** Mean absolute SHA*p* value bar plot ranking global feature importance. **(C)** Waterfall plot explaining an individual prediction. **(D)** Force plot illustrating the additive feature contributions for another individual prediction.

Due to its superior performance, SHAP analysis was performed to interpret the model’s predictions and elucidate the contribution of individual features. The global feature importance for PHN prediction, derived from SHAP values, was ranked in descending order as follows: age, timing of antiviral therapy, acute pain severity, prodromal pain, and diabetes. This ranking quantifies the relative contribution of each predictor to the model’s output, as detailed in Figure 5.

## 4 Discussion

Despite guideline-concordant antiviral and analgesic management, the incidence of PHN in this study remained substantial, reaching 19.0% in the training cohort and 22.8% in the external validation cohort, highlighting a persistent clinical challenge. The pathogenesis primarily involves direct viral injury to sensory ganglia and the consequent neuroinflammatory cascade during acute infection. Specifically, reactivation of VZV triggers ganglionitis and axonal degeneration, processes that drive both peripheral and central sensitization alongside maladaptive neuroplasticity (32, 33). These alterations lead to sustained dysfunction within nociceptive pathways, manifesting as the characteristic chronic neuropathic pain that persists long after rash resolution. As outlined in contemporary pathophysiological models, key mechanistic contributors include central sensitization (mediated by neurotransmitters such as glutamate and substance P), peripheral sensitization (involving upregulation of voltage-gated ion channels), spinal deafferentation, and ectopic impulse generation in dorsal root ganglia (34–36).

Machine learning offers a powerful approach for clinical prediction by capturing complex, non-linear relationships among variables (37). In this context, we systematically evaluated 10 machine learning algorithms to identify the optimal model for predicting PHN. The XGBoost model demonstrated the most robust and consistent performance. Integration of this model into clinical practice, for instance as a decision-support tool within electronic health records, could enable early risk stratification following an HZ diagnosis. This early identification creates a critical window for implementing a comprehensive management strategy. For high-risk patients, this may include not only optimized analgesic regimens and consideration of interventional procedures, but also timely referral for emerging non-pharmacological therapies. Promising avenues in this regard include electroacupuncture and moxibustion, which have shown efficacy in alleviating PHN in recent studies (38, 39). Furthermore, the ongoing exploration of novel predictive tools, such as infrared thermography, may complement clinical prediction models in the future, enriching the toolkit for early and personalized intervention (40).

The SHAP analysis identified age as the most influential predictor, underscoring its central role in PHN pathogenesis. This finding aligns with established epidemiology showing a marked increase in both HZ incidence and PHN complications among older adults, a phenomenon largely driven by age-related immunosenescence (25, 41). Immunosenescence involves a progressive decline in cell-mediated immunity, particularly T-cell function, which impairs the containment of VZV reactivation and mitigation of subsequent neural damage. Consequently, elderly patients are more susceptible to prolonged viral replication, axonal degeneration, and central sensitization—all processes that heighten the risk of chronic neuropathic pain (42).

Initiation of antiviral therapy more than 3 days after rash onset emerged as a significant risk factor for PHN development. This finding aligns with current guidelines that emphasize a critical window for initiating antiviral therapy within 72 h of vesicle emergence to maximize efficacy (7). The rationale for early intervention lies in the rapid suppression of VZV replication. By reducing the viral load during the acute phase, antiviral agents limit the extent of nerve inflammation, hemorrhagic necrosis, and subsequent axonal degeneration within sensory ganglia. Minimizing this initial neuronal injury is pivotal to preventing the cascade of peripheral and central sensitization underlying the persistent neuropathic pain characteristic of PHN (20, 23, 43).

The severity of acute pain at HZ onset was also a significant predictor of PHN. Mechanistically, greater acute pain intensity reflects more severe neuroinflammation and more extensive neuronal injury within the affected dermatome. This pronounced inflammatory milieu not only exacerbates peripheral sensitization but also drives more robust and sustained central sensitization at the spinal and supraspinal levels. Furthermore, acute pain intensity may serve as a clinical indicator of higher viral burden or a more vigorous host inflammatory response, both of which contribute to amplified and prolonged damage to sensory nerves and ganglia (21, 44).

Prodromal pain, occurring before the vesicular rash, was also a significant predictor of PHN. This early symptom—often described as tingling, burning, or hypersensitivity in the affected dermatome—signifies the initial reactivation and replication of VZV within sensory neurons. Pain at this stage indicates that significant neural inflammation and subclinical neuronal injury are already underway, preceding visible skin lesions. This early neural insult can trigger a more profound inflammatory cascade within the dorsal root ganglion and peripheral nerves, establishing a state of peripheral and central hyperexcitability prior to overt clinical manifestation. Consequently, when the rash appears, the nervous system is already primed for a heightened and prolonged pain response, thereby increasing susceptibility to persistent neuropathic pain (22, 41, 43).

Diabetes mellitus was confirmed as a significant risk factor for PHN, consistent with prior evidence. The elevated risk is attributed to the multifaceted impact of diabetes on both immune function and neural integrity. Chronic hyperglycemia impairs innate and adaptive immune responses, including reduced T-cell activity and neutrophil function, which compromises the containment of VZV reactivation during acute HZ (45, 46). Furthermore, diabetes predisposes patients to peripheral neuropathy and microvascular complications, which amplify inflammatory neural damage, delay tissue repair, and promote central sensitization pathways that facilitate pain chronification (47–49).

Regarding the site of involvement, whether HZ affecting the head and neck inherently confers a higher risk of PHN compared to other sites remains debated in the literature (10, 19, 20, 50). In our feature selection process, the variable “Site of involvement” was explicitly evaluated but was ultimately rejected by both the LASSO regression and the Boruta algorithm due to its insufficient independent predictive power relative to other clinical factors. The exclusion of this variable from our final model can be interpreted through two clinical considerations. First, head and neck involvement is frequently associated with more severe acute pain (51). This clinical characteristic is likely captured by the variable “Acute pain severity”, which was itself a significant predictor in our final model. Second, due to the recognized risk of serious complications (e.g., ocular involvement), HZ in the head and facial regions is typically managed more aggressively, including earlier and more intensive antiviral and analgesic therapy. Such proactive management may have mitigated the site-specific risk of progressing to PHN.

Several limitations of this study should be acknowledged. First, the retrospective analysis of prospectively collected data may introduce selection and information biases. Second, although external validation was performed, the validation cohort originated from a single center within the same province, which may restrict the model’s generalizability to broader or more diverse populations (external validity). Additionally, potential unmeasured confounders, such as detailed treatment regimens, vaccination status, or psychosocial factors, could not be adjusted for and may influence the model’s predictive accuracy in real-world clinical practice.

## 5 Conclusion

We developed and externally validated an XGBoost-based prediction model that effectively stratifies PHN risk using five readily accessible clinical factors. The model demonstrated robust performance and interpretability, providing a practical and interpretable tool to support early clinical decision-making for patients with herpes zoster.

## Data Availability

The original contributions presented in the study are included in the article/Supplementary material, further inquiries can be directed to the corresponding author.
