# Supplementary material for: Development and validation of a machine learning model for predicting postherpetic neuralgia risk
Source: Front Neurol. 2026 Apr 16;17:1775957. doi: 10.3389/fneur.2026.1775957 (PMC13128426; doi:10.3389/fneur.2026.1775957)
Supplement: Supplementary file 1 [file Table_1.DOC]

**Supplementary Table 1. Hyperparameter search spaces explored in grid search with 5-fold cross-validation.**

| Model | Hyperparameters and Search Ranges |
| --- | --- |
| RF | n_estimators: [100, 200]; max_depth: [5, 10, None]; min_samples_split: [2, 5] |
| XGBoost | learning_rate: [0.01, 0.1]; max_depth: [3, 6]; n_estimators: [100, 200] |
| LightGBM | num_leaves: [31, 63]; learning_rate: [0.01, 0.1]; n_estimators: [100, 200] |
| AdaBoost | n_estimators: [50, 100]; learning_rate: [0.1, 1.0] |
| SVM | C: [0.1, 1, 10]; kernel: ['linear', 'rbf'] |
| LR | C: [0.1, 1, 10]; penalty: ['l1', 'l2']; solver: ['liblinear'] |
| ANN | hidden_layer_sizes: [(64,), (128,), (64, 32)]; alpha: [0.0001, 0.001]; max_iter: [500] |
| NB | var_smoothing: [1e-9, 1e-8, 1e-7] |
| KNN | n_neighbors: [3, 5, 7]; weights: ['uniform', 'distance']; metric: ['euclidean', 'manhattan'] |
| DT | max_depth: [5, 10, None]; min_samples_split: [2, 5]; criterion: ['gini', 'entropy'] |

**Supplementary Table 2. Hardware configuration and computational time for model training.**

| Component | Specification / Description |
| --- | --- |
| Central Processing Unit (CPU) | Intel Core i7-12700H |
| Graphics Processing Unit (GPU) | NVIDIA GeForce RTX 3060 Laptop GPU (6GB VRAM) |
| Memory (RAM) | 16 GB DDR4 |
| Software Platform | R 4.5.2, Python 3.9 (with scikit-learn, XGBoost libraries) |
| Average time per model training cycle | 2-8 minutes (varies by model complexity) |
| Total time for full training & evaluation of ten models | 1.5 hours |
